# Food habits of different worker categories: an integrative review

**DOI:** 10.47626/1679-4435-2022-703

**Published:** 2023-02-13

**Authors:** Antônia Flávia Lopes de Sousa, Raquel Teixeira Terceiro Paim

**Affiliations:** 1 Nutrição, Centro Universitário Unifametro, Fortaleza, CE, Brazil

**Keywords:** occupational health, eating habits, work, saúde ocupacional, hábitos alimentares, trabalho

## Abstract

Food is a physiological necessity for humans and is built on and permeated by many different biological, economic, social, and cultural symbols and phenomena. The basic conditions for adequate nutrition should be associated with cultural and financial values, physical accessibility, flavor, variety, color, and harmony and based on consumption of foods, not exclusively on nutrients. However, changes to the population’s profile of consumption and dietary habits are founded on the process of urbanization and industrialization, which plays a fundamental role in this phenomenon, causing lifestyle changes that are linked with stimulus of consumption of industrialized products, with publicity, and with mass marketing. The objective of the study was to investigate the profile of the dietary habits of workers from different occupational categories in Brazil, with a sample of 13 articles. Moreover, research shows that many different categories of workers are subject to nutritional losses because of this new lifestyle. Searches were run on the Google Scholar, LILACS, and SciELO databases for publications during the last 5 years, identifying more than 15 thousand articles, 13 of which were selected as fitting the criteria chosen. Data were collected in April and May of 2020. The inclusion criteria were articles published in Portuguese with the full text available. Exclusion criteria were duplicates and studies with seniors and/or children. It was concluded that the dietary habits of the workers studied are unhealthy and that their consumption profile is widely incompatible with the guiding principles of the Food Guide for the Brazilian population. These people are therefore at increased risk of non-transmissible chronic diseases and morbidity and mortality. There is a need to take more effective interventional action, totally restructuring the educational process to form dietary habits, such as implementing public policies targeting this section of the population, which is so important for national development.

## Abstract (pt)

A alimentação é uma necessidade fisiológica humana, construída e permeada por diversas simbolizações e fenômenos biológicos, econômicos, sociais e culturais. A condição básica para uma alimentação adequada deve estar associada aos valores culturais e financeiros, à acessibilidade física, ao sabor, à variedade, à cor e à harmonia, com base no consumo de alimentos, e não exclusivamente de nutrientes. Porém, a mudança no perfil de consumo e nos hábitos alimentares da população está alicerçada no processo de urbanização/industrialização, que atua como fator preponderante para esse fenômeno, gerando mudanças no estilo de vida associadas ao estímulo ao consumo de produtos industrializados, à publicidade e ao *marketing* massivo. Nesse contexto, o objetivo deste estudo foi investigar o perfil dos hábitos alimentares de trabalhadores de diferentes categorias profissionais no Brasil, com 13 artigos selecionados. Além disso, pesquisas apontam para o fato de que trabalhadores de diferentes categorias estão sujeitos a perdas na alimentação devido ao novo estilo de vida. Foi realizada uma busca nas bases de dados Google Acadêmico, LILACS e SciELO de publicações dos últimos 5 anos, e foram encontrados mais de 15 mil artigos; destes, foram selecionados 13 que se encaixam no padrão escolhido. A coleta dos dados ocorreu em abril e maio de 2020. Os critérios de inclusão foram artigos disponíveis na íntegra e em língua portuguesa; já os de exclusão foram trabalhos duplicados ou com idosos e/ou crianças. Concluiu-se que os hábitos alimentares dos trabalhadores pesquisados são inadequados e que esse perfil de consumo está em desacordo com os princípios norteadores defendidos pelos Guias Alimentares para a população brasileira, de forma ampla e difusa. Assim, tais indivíduos apresentam maior risco de sofrer doenças crônicas não transmissíveis e de apresentar morbimortalidade. Urge a necessidade de ações intervencionistas mais efetivas para uma reestruturação total do processo de educação para a formação de hábitos alimentares, como as políticas públicas voltadas para essa classe civil, tão importantes para o desenvolvimento de uma nação.

## INTRODUCTION

Food is a physiological necessity for humans that is built on and permeated by many different symbols and biological, economic, social, and cultural phenomena, and is essential for human growth and development.^1^

A healthy diet is not simply a matter of eating healthy preparations; there are also practical peculiarities that are founded on the principles of prevention and health promotion. The basic conditions for adequate nutrition should be associated with cultural values, with physical and financial accessibility, with flavor, variety, color, and harmony, and should be guided by consumption of foodstuffs, not exclusively based on nutrients.^2^

Over recent decades, the population’s nutritional profile has shifted, with changes in its characteristics of food consumption and dietary habits, provoked by the process of urbanization and industrialization, which plays a fundamental role in this phenomenon, causing changes to lifestyle and to health that are linked with stimulus of consumption of products, food industry advertising, and mass marketing.^3^

This scenario results in nutritional imbalances, such as insufficient dietary intake, causing nutritional deficiencies and excessive intake, resulting in excess weight and obesity, which are ever more prevalent in society. The second of these phenomena has provoked the rise of chronic degenerative diseases globally, particularly cardiovascular diseases, diabetes mellitus, hypertension, and different types of cancer - all caused by poor dietary habits and a harmful lifestyle, among other factors.^4^

In view of this, and in response to the alarming situation with regard to emergence of chronic diseases in the Brazilian population, the World Health Organization (WHO) has published a Food Guide with the primary objective of promoting the health of the Brazilian population through recommendations and information on healthy eating, in addition to preventing non-transmissible chronic diseases (NTCD), helping to control weight, while improving the body’s functioning and providing it with essential nutrients.^5^

This instrument inspired and supported development of questionnaires based on portions of foods classified by food groups, to be used to categorize consumption profiles and indicate potential risk of health problems cause by the diet. The methods based on these guidelines are intended to survey the presence of different food groups, assessing the daily intake of each group, with the objective of identifying the current status of consumption and highlight the importance of variety and of intake of nutritional components.^6^

Additionally, the guide is also oriented by the principal of improving people’s diets through the Workers’ Food Program (WFP), which is specifically tailored for workers. It has been used to improve workers’ nutritional status, with positive consequences for quality of life and improvements in productivity and wellbeing, achieving a healthy dietary intake profile and supporting growth in this category of workers’ lives.^7^

However, research has shown that workers are vulnerable to compromised nutrition caused by the characteristics of their jobs, just as the population in general is vulnerable to the effects of the new lifestyle adopted, while the level of efficiency that people are able to achieve at work and the tasks they can fulfill depend on their health status. Moreover, excessive consumption of calories can have disastrous consequences over the long and short term, such as reduced performance at work and increased risk of accidents in the workplace.^8^

In view of the above, the objective of this study was to conduct a literature review to investigate the dietary habits of different categories of workers in Brazil.

## METHODS

An integrative literature review was conducted of research into the profile of workers’ dietary habits, aiming to summarize and analyze the dietary intake of the study population, using the following guiding question: “What is the profile of Brazilian workers’ dietary habits?”

Parallel searches were run on the databases Google Scholar, Latin American and Caribbean Health Sciences Literature (LILACS), and the Scientific Electronic Library Online (SciELO) for publications from the last 5 years. The keywords used were “dietary habits”, “workers”, “dietary profile”, and “workers’ nutrition”.

The inclusion criteria were cross-sectional, observational, and descriptive studies published in Portuguese for which the full text was available. Studies were excluded that were duplicates or literature reviews and those with elderly and/or pediatric samples.

Approximately 15,000 articles were found on Google Scholar, 33 on SciELO, and 100 on LILACS. These were winnowed down to those that specifically reported the dietary habits of workers from different occupational categories; resulting in selection of 13 articles that met the criteria established.

Articles were selected in April and May of 2020 by a researcher conducting separate searches. A critical and detailed analysis was conducted, comparing the data with theoretical knowledge and the healthy eating guidelines, identifying conclusions on the profile of these populations’ dietary habits.

## RESULTS

Chart 1 summarizes the articles selected for the review, listing their lead authors, locations, year of publication, participants, objectives, study designs, and principal conclusions.

**Chart 1:** Distribution of the references selected for the systematic review, by lead author/year/location, participants, objective, study design, and principal conclusions

N.°	Author/year/location	Study participants	Research objectives	Study design	Principal conclusions
1	Freitas et al.^9^/2015/Rio de Janeiro	Meat-packing plant workers	To investigate the influence of shift work on the eating behavior of workers at a meat-packing plant in the South of Brazil.	Cross-sectional	It was found that the workers most often ate three meals a day and that it was predominately older workers who ate healthy foods. Younger workers had a poor diet, characterized by consumption of pre-prepared food.
2	Falcão et al.^10^/2015/Rio de Janeiro	Workers at “Popular Restaurants”	To analyze the prevalence of perceived food insecurity in the households of “Popular Restaurant” workers, and analyze associations between this perception and socioeconomic, occupational, and health variables.	Cross-sectional	Almost half of the participants eat fruit and vegetables daily and are satisfied with the composition and regularity of their diets at the workplace, but the quantity of fruit, vegetables, and greens eaten at home is insufficient because they do not eat them daily or in sufficient quantity.
3	Santos et al.^11^/2016/São Paulo	Bus drivers	To analyze the dietary habits and diet quality of bus drivers.	Cross-sectional	It was observed that the bus drivers consume insignificant quantities of fruit, vegetables, greens, and milk and dairy products and large quantities of meat in few meals per day, adding up to a maximum of three: breakfast, lunch, and dinner.
4	Freitas et al.^12^/2016/Minas Gerais	Municipal public sector workers	To determine whether sociodemographic characteristics, dietary habits, health status, and working conditions are related to excess weight in municipal public sector workers.	Cross-sectional	It was observed that lack of time to prepare meals caused excessive intake of fats and sodium, because of high levels of consumption of foods containing skin and fats and of salt added to prepared food. Less than half (48.5%) of the sample regularly eat fruit and salad vegetables.
5	Rizzi et al.^13^/2017/Rio Grande do Sul	Postal workers (postmen)	To assess the nutritional status and dietary intake of postmen who work on foot in Porto Alegre.	Cross-sectional	There were high levels of consumption of red meat, sodas, and artificial juice, leading to high intakes of sodium and copper, and low consumption of fish, omega 3 and 6 fatty acids, and the minerals potassium and magnesium. No consumption was observed of essential foods such as fruit, greens, and vegetables, making their diets poor and unhealthy.
6	Braga et al.^14^/2017/Amazonas	Workers from the Nutrition and Dietary Service at a regional hospital	To assess the workers’ nutritional status, dietary habits, and health status.	Descriptive, exploratory	It was concluded from the dietary intake statements that the foods most eaten were meat, milk, pulses, fruit, and salad vegetables, supporting adequate nutritional status, and providing sufficient energy for those working. However, the quantities need to be adjusted. Although these workers eat a good variety of foods, the quantities eaten are not in accordance with recommended parameters.
7	Artuzo et al.^15^/2017/Paraná	Hospital workers	To analyze the clinical and nutritional features of a hospital unit.	Cross-sectional	The study observed adequate intake of carbohydrates, from 20 to 90%, 100% for proteins, and 20 to 80% for lipids. The most prevalent frequency of consumption was 1 to 3 times per week in both shifts for the following food groups: sausages/salami, snacks, fried food, milk and dairy, cakes, sweets, and desserts, and sodas and industrialized juices.
8	Rodrigues et al.^16^/2018/Paraná	Night shift workers at a hotel chain	To analyze the dietary habits of night shift workers at a hotel business	Observational, descriptive	Intake of all food groups was inadequate, with high intake of red meat and sausages/salami. Swapping day for night causes problems, resulting in insufficient daily intake of meals and nutritional components.
9	Tartari et al.^17^/2018/Rio de Janeiro	Workers at a fast food chain	To illustrate workers’ lifestyle changes and analyze and compare dietary habits and health before and after induction to the job, in order to determine whether employment imposed changes to nutritional routine.	Observational, longitudinal, and retrospective	The study observed elevated consumption of hamburgers, fries, and sodas, food with high calorie content and dubious nutritional value, such as that at the fast-food chains at which they work.
10	Thumé & Poll^18^/2018/Rio Grande do Sul	Public sector workers	Determine the proportion of healthy eating among adult public sector workers.	Cross-sectional	The study population had unhealthy diets, characterized by low intake of food groups (fruit, greens, vegetables, and pulses) and high intake of animal fat, simple sugars, and salt.
11	Sanchi & Borges^19^/2019/Rio Grande do Sul	Bank workers	Describe the lifestyle and nutritional status of workers at a banking chain.	Cross-sectional, descriptive	Unhealthy dietary habits were observed, with high levels of sweetened and fatty foods and low intake of fruit and vegetables, in addition to sedentary behavior in 67% of male interviewees, indicating an unhealthy lifestyle.
12	Schäfer et al.^20^/2019/Santa Catarina	University workers	Analyze the workers’ dietary intake.	Cross-sectional	It was concluded that the participants had low frequency of consumption of foods considered markers of healthy nutrition, such as fruit, greens, vegetables, and pulses. Additionally, many of them stated they had unhealthy eating habits, such as eating while watching television.
13	Cattafesta et al.^21^/2019/Espírito Santo	Bank workers	To analyze the dietary intake of bank employees using principal components analysis and identify associations with socioeconomic factors and behavioral and occupational characteristics, to identify factors associated with the dietary intake of workers with intense shifts and workloads.	Observational, cross-sectional	Three patterns of dietary intake with high frequencies were identified: “salad vegetables, fruit, cereals, and root vegetables”, “sweets and snacks”, and “traditional and protein-based”. Food choices were influenced by time and availability of foods.

All 13 studies were conducted in Brazil and aimed to analyze the lifestyle profiles of workers from a range of different occupations, in some case revealing the perception of a relationship associating employment with changes to the ideal dietary profile and to workers’ nutritional status.

With relation to sample size, the numbers of participants ranged from 20 to 1,206, which proved appropriate for the type and method of the study, since the cross-sectional and observational studies employed questions for identification of intake profiles that were similar to what is found in the Food Guide for the Brazilian population,^22^ without changing or manipulating the natural environment, making it possible to compare different samples from different periods in time (2015 to 2020) and workers from different age groups (20 to 50 years).

From this perspective, the objective of the studies listed in Table 1 was to employ cautious methods that enable increased verification of the variables studied, supporting valid repercussions that respond better to identify the profiles of the workers’ dietary habits. These studies were therefore based on dietary surveys, such as 24-hour recall and food frequency questionnaires, which demonstrated that the study population’s intake and daily frequency of consumption of some food groups were insufficient.

From this angle, use of food questionnaires as a rigorous method enables greater mastery of the study variables, objectively revealing the frequency and characteristics of consumption, which were found not to comply with Ministry of Health recommendations.

It was also notable that the study revealed inadequate consumption of essential nutrients, characteristic of diets with low consumption of fruit, greens, vegetables, and pulses, and elevated consumption of red meat, industrialized foods, sausages/salami, sweets, simple sugars, and fats, in particular among the postmen, bus drivers, and bank workers.

In some studies, the consumption profile was explained by lack of time and the ease of preparation of industrialized meals, leading to food choices characterized by high calorie content and low nutritional value, with inadequate intake of the majority of the food groups that are necessary to maintain the body healthy.

## DISCUSSION

Development and the advance of industrialization, of urbanization, and of globalization that have taken place over recent years have provoked excessive social and economic changes in many countries. The result has been a gigantic change in people’s lifestyles, with emphasis on important changes in nutritional aspects and linking an unhealthy diet, characterized by the constant presence of industrialized foods, rich in fat, sugar, and salt, to inactivity.^23^

In view of this, a process known as nutritional transition has taken hold in modern times, by which a profile dominated by malnutrition has given way to a growing and epidemic expansion of a nutritional profile consisting of excess weight and obesity, exacerbating multifactorial conditions through interaction between genes, lifestyle, and environment and emotional factors, becoming a well-known risk for emergence of DCNTs. These conditions include many different health problems, including diseases such as arterial hypertension, diabetes mellitus, coronary diseases, myocardial infarction, and some types of cancer.^24^

It should be emphasized that diet is one of the reasons for healthy development and function of the body, since nutrients are essential for maintenance and growth of tissues and are responsible for providing energy, in addition to supporting adequate performance at work and the ability to fulfill job tasks.^1^

In view of this, the present study attempted to understand the characteristics of the eating habits of workers from a wide range of occupations in terms of their diets, taking into consideration that dietary habits are built up through daily customs and are subject to considerable influence from the social group and working environment, to which a significant part of people’s time and lives are linked.^15^

Almost all of the 13 studies identified in the analysis of the literature on the subject identified inadequacies of daily dietary intake (contrasting, in some respect with what is recommended by health authorities) and an inadequate distribution of meals over the course of the day (one to three). It was therefore shown that the entire population analyzed had unfavorable dietary habits.

This portrait is confirmed by the results reported in the most recent Family Budget Surveys (FBS)^25^ conducted in Brazil, based on surveys conducted in 2002-2003, 2008-2009, and 2017-1018, which showed that foods eaten unprocessed or minimally processed and culinary ingredients are being replaced by processed foods and, even more so, by ultra-processed foods. This finding is of concern, because it is in direct conflict with the recommendations of the Food Guide for the Brazilian population, which recommends the exact opposite - i.e. that a good diet should be based on foods eaten unprocessed or minimally processed and their culinary preparations and that ultra-processed foods should be avoided.

To provide values for comparison and a model for guidance on the national level, the Food Guides for the Brazilian population recommend that people eat at least three meals per day (breakfast, lunch, and dinner) and two or three snacks between main meals. This is an attempt to ensure the health of the population, encouraging them to adopt and maintain healthy eating habits appropriate for each cycle of life.^5,22^ However, the majority of the workers in the occupational categories analyzed in this study do not comply with the instructions contained in the guidance documents, failing to meet the recommendations for intake of foods in the food groups and consuming elevated quantities of foods that are not recommended for achieving good health.^26^

The 2006 Food Guide for the Brazilian population states that the portions of each food group should be distributed according to the bioavailability of their most prevalent nutrient, categorized as cereals, tubers, root vegetables and sub-products; fruit; greens and vegetables; pulses; milk and dairy; meat and eggs; sweets and sugars; and fat, in addition to recommending moderate consumption of salt, sufficient intake of water, and remaining physically active. The objective of the process is to promote wellbeing among the Brazilian population, reducing the risk of events with avoidable causes that are prejudicial to health and can result in high rates of morbidity and mortality.^22^

This is important because the nutrients contained in unprocessed foods, such as fruit, greens, and vegetables contain substances with the potential to protect our bodies against NTCDs and which, when eaten daily, are markers of a healthy and balanced diet.^20^

Therefore, compared with a study of dietary intake in Brazil conducted by Canuto et al.,^27^ the results of this study confirm an unhealthy scenario in which variables such as economic development allied to the nutritional transition process have impacted and remodeled Brazilians’ eating habits, predominantly those of the less privileged socioeconomic classes. The social result of these changes is a diet that incorporates the unhealthy combination of foods that have high calorie content, but are poor in nutrients, which, as a consequence, leads to population profiles at risk of ill health.

Additionally, it should be highlighted that there are programs in our country that specifically target the working population, in particular the WFP within the nutritional sphere,^7^ which is intended to improve these people’s nutritional status, leveraging diet to achieve results compatible with improvement of quality of life, meeting their nutritional requirements in proportion to the duration of their work shifts, offering nutritious and balanced meals, reducing the likelihood of diseases associated with nutrition, and enriching the state of public health.^28^

In general, this study has also shown that these workers predominantly have an unhealthy and disorganized meal pattern, in addition to eating foods that are easily accessible and quick to prepare, with a high frequency of consumption of sausages/salami, industrialized foods, artificial juices, sodas, fats, and sugars.^29^

Resulting from this unhealthy diet, there is a great potential for the emergence of certain high-risk conditions that are provoked by the change in lifestyle, such as diseases that may be diagnosed over time. Although chronic diseases have proven persistent, with a trend to worsen and/or to relapse events, it should be emphasized that, once they have been diagnosed, cultivation or incorporation of factors that are protective of these people’s lives can substantially reduce the risks or severity of these diseases.^30^

This stated, it is important to point out/emphasize that the indications presented here confirm the need for an intervention in this area, in the institutional and governmental spheres, in view of the importance of this social stratum to development of the country, in addition to the need for awareness of the potential social, economic, and affective harm of failure to act.

As an important tool for transformation, nutritional education can be an important proposal that targets health promotion and avoidance of the risks that diseases can impose by means of modifying unhealthy eating habits and sensitizing people to making healthy eating choices. The nutritionist is the professional responsible for nutritional and dietary progress who is best trained for this task, helping to spread these healthy habits.^31^

It is also clear that the workplace is an appropriate setting for health education activities, which can be conducted by the organizations for which these employees work. It is therefore of interest to develop new preventative approaches for these groups that can contribute to improving their health, performance at work, and enjoyment.

Limitations of this study include the small number of studies in the area and the fact that all of those reviewed used methods that were approximate or had the same objective.

## CONCLUSIONS

This study found that in almost all of the articles reviewed, the dietary habits of workers were found to be unhealthy and that although consumption of the essential nutrients present in food groups such as fruit, greens, and vegetables was relatively frequent, they were not consumed in the ideal quantities to supply the population’s needs, and neither their breadth nor reach were satisfactory according the recommendations of the Food Guide for the Brazilian population.

It should also be emphasized that the working environments of those studied may have contributed negatively to construction and consolidation of these habits, which combine a collection of worrying and unhealthy factors, such as elevated consumption of industrialized food, sausages/salami, sugars, and fats, typical of a poor diet and also characteristic of a lifestyle that has spread and become established in Brazil. There is a serious need for care measures, guidance, and strategies focused on food and nutrition that act in coordination to support the wellbeing of workers.
